# Nervous Diseases

**Published:** 1893-05

**Authors:** James W. Putnam

**Affiliations:** Buffalo, N. Y.


### Progress in Medical Science.

### NERVOUS DISEASES.

By JAMES W. PUTNAM, M. D., Buffalo, N. Y.

### PROGRESSIVE MUSCULAR ATROPHY.

ZENNER (*Alienist and Neurologist*, January, 1893). In an able paper the author analyzes the symptoms and clinical history of the three types of progressive muscular atrophy—the spinal, muscular, and neuritic.

The spinal, or the Duchenne-Aran type, occurs mostly in middle adult life, most frequently beginning in the small muscles of the hand, and attacks successively other groups of muscles. Its march is very slow. There is atrophy, paralysis, and altered electrical reaction. The reaction is rarely one of complete degeneration.

The muscular form is known as Erb's juvenile atrophy. This type includes the form which usually begins in the muscles of the upper arm and shoulder. It sometimes, also, includes the muscles of the back, and lower extremities. In some cases some muscles are found hypertrophied, notably the deltoids, the infra-spinati, triceps, sartorius, and the muscles of the calf.

It will be seen that Erb puts in the same class juvenile atrophy, pseudo-hypertrophic paralysis, and the facio-scapulo-humeral type of muscular atrophy. These are grouped into the one disease under the term of progressive muscular dystrophies. Of this class the author quotes Erb thus: "Even Erb, in his latest publication on the subject, has expressed the opinion that the muscular disease is due to the changes in the ganglion cells of the anterior cornua, but changes so slight that the microscope, as yet, does not reveal them."

Of the peroneal type, which is also a disease of early life and, more or less, a hereditary one, he acknowledges that its distribution is the same, as is common in most cases of multiple neuritis, but he believes, with Hoffman, that the neuritis in these cases is secondary to disease of the large ganglion cells in the cornua.

He, therefore, concludes that there is reason to believe that these various forms of muscular atrophy really are due to disease of the anterior cornua, whether that disease be gross or so inappreciable to the eye, that it is termed functional.

---

### ITCHING OF CENTRAL ORIGIN, OR BRAIN ITCH.

BREMER, (*Review of Insanity and Nervous Diseases*, December, 1892,) arguing from Edinger's case of paralysis of the right arm and leg, accompanied by athetosis and by excruciating pain at all times, in whom was found a softened spot in the external nucleus of the left thalamus opticus, accepts the theory that there may be pain of central origin. For how else, he says, will you explain the pain of the hypochondriac or the hysterical, or that of hypnotic suggestion ? The epileptic aura, which may be an itching instead of a pain, he explains as a projection of a cortical irritation on a corresponding area of the skin. He refers to the well-known fact that certain persons cannot hear vermin spoken of without being afflicted with a sensation of itching, which, he says, is brought about by an association process in the hemispheres. He says he has often seen pruritus accompany melancholia, and reports two cases in which it preceded the mental trouble. The pruritus that is so frequently met with in hysteria and neurasthenia is, in his opinion, undoubtedly, a brain itch, and not a peripheral one. He makes a strong plea against the custom of gynecologists of treating pruritus about the vaginal orifice in neurasthenic women.

For treatment he recommends combining bromide (10 to 12 grains) and cannabis Ind. ext. ($\frac{1}{4}$ gr.), three times daily.

He also advocates a warm bath, with a handful of wash soda and a half pound of starch, in an ordinary bathtubful of warm water. Of this he says :

> This simple measure has given better satisfaction than any other in institution treatment when such cases are notoriously often a source of despair both to the physician and patient. Even in strictly central pruritis it generally acts well, owing, probably, to the sedative effect which the warm bath has on the cortex.

---

### HYPNOTICS.

Kaumheimer (*The Review of Insanity and Nervous Diseases*, December, 1892). In an abstract from other authors Kaumheimer quotes :

So much can be said in regard to chloralamid that the conclusion is forced upon us that in this substance we have an agency for the production of sleep which is deserving of our most sanguine expectations. Chloralamid can be used in cases where chloral cannot be used, as, for instance, where there is weak heart in the asthenic stage of acute disease.

On the action of trional and tetronal he summarizes from Schaefer :

1. Trional and tetronal are of pronounced hypnotic and sedative action. Tetronal seems to be more sedative than trional. The action begins within ten to twenty minutes.

2. Trional is a certain and prompt hypnotic, and is indicated in the different forms of neurasthenia, functional psychoses, and organic brain troubles. It does not relieve pain, and is useless in cases of morphinism and cocainism.

3. Tetronal is indicated as an hypnotic in cases in which a moderate degree of motor excitement prevents sleep. Neither can be recommended in the severer psychoses, with violent motor excitement. Doses are from one to two grammes.

No unpleasant effects have been noticed either during administration or after withdrawal. (The great objection to these drugs is their cost : Trional costs a trifle more than sulphonal, and tetronal costs twice as much as trional.)

---

### THE BLOOD IN MELANCHOLIA AND THE EFFECT OF SYSTEMATIC TONIC TREATMENT.

Steele (*American Journal of Insanity*, April, 1893). A table of thirty-five cases of melancholia shows that anemia was quite marked in most of the cases, and that the average of red corpuscles and of hemaglobin was much below normal. Only one case showed a normal amount of corpuscles and hemaglobin.

Twelve of these cases were selected and put on a special tonic treatment, which included cod liver oil, quinine, and strychnia.

After six weeks the blood was again examined. In six of the cases there was no improvement, either in the quality of the blood or in the mental symptoms. In the other six cases there was improvement in both respects. He draws the following conclusions: That systematic tonic treatment is found markedly efficacious in the treatment of melancholia. The administration of iron by itself, or a combination of iron, quinine, and strychnia, seems to be equally effective. It would appear, also, that although melancholia may not be caused by an impoverishment of the blood, *per se*, such impoverishment almost invariably exists, and, in a large majority of cases, improvement of the mental symptoms is coincident with improvement in the general health and in the quality of the blood.

---

### TWO CASES OF VERY LARGE TUMORS OF THE CEREBRUM.

(*Berlin Klin. Wochenschrift*, No. 29, 1892.) There were two cases operated on by Prof. von Bergmann. One was a mixedsarcoma, and weighed 280 grammes, or nearly half as much as the weight of the cerebral hemisphere, which was assumed to be about 640 grammes. This was shelled out from the right frontal lobe.

Case two was that of a man, aged forty-seven, who had received a blow on the head eighteen months before. The symptoms were those of a tumor of the right motor region. A cystic tumor, which was a portion of an infiltrating cysto-sarcoma, was removed. The tumor was the size of a duck's egg. In addition to this, large masses of the tumor were removed at two subsequent operations. Six months afterward both patients were reported alive.

---

### MOTOR NEUROSES OF THE HEART.

DR. JACOBY, (*New York Medical Journal*, April 8, 1893,) in an analytical study of this subject—arrhythmia—bradycardia and tachycardia are considered. When arrhythmia is temporary, he believes it to be either a pure neurosis or dependent upon defective blood mixture. If a pure neurosis, it may be a reflex from an irritated stomach or intestines; frequently it is a part of general neurasthenia. It is often due to psychic influences of a depressing nature. Of all causes which he has found most potent in the production of arrhythmias are toxic influences, in the excessive use of tea, coffee, tobacco, and alcohol, associated with sexual excesses.

Of slow heart, or bradycardia, he says: "Whether bradycardia ever occurs as a pure neurosis, in the same sense as paroxysmal tachycardia does, is more than doubtful."

In his discussion of tachycardia, he excludes cases of a symptomatic nature, and considers only those of violent heart's action, which occur paroxysmally and without obvious reason. In these cases the patient feels perfectly well in the interparoxysmal period, and no cardiac disease can be discovered. Of its etiology, all that he positively ventures is, that it is a disorder of adult life, and that the best established causes are bodily and cerebral overwork. He does not consider that nervous predisposition, as hysteria and neurasthenia, has any particular influence upon its production.

As to the nature of the affection, he commits himself to the theory that it is a bulbar neurosis; that a case which may begin as a pure neurosis may later become one of organic disease.

He maintains that neuroses are often precursors of organic disease ; or, to put it more strongly, that organic disease is sometimes the direct outcome of a functional disorder.

So, in all cases of motor neuroses of the heart, what today is looked upon as a pure neurosis may remain so for a period of time and then get well, or may develop into organic disease. As long as repair outbalances waste, the disease remains functional, and when waste exceeds repair, then we have lesions and organic disease.

---

### EYE PARALYSIS.

JEFFRIES (*Boston Medical and Surgical Journal*, October 26 and 27, 1892.) The author sums up his conclusions as follows :

1. All cases of lateral conjugate paralysis are of central origin.

2. When the paralysis is on the same side as other paralyses, the lesion is on the opposite side of the brain. Such paralyses, as a rule, are transitory, and follow almost any sudden lesion, and often show themselves as a prevailing position of the eye, and not as a true paralysis or even paresis.

3. When the paralysis is crossed with the paralysis below, the lesion is in the pons-medulla region. The above three are equally true of spasms.

4. A gradual development of conjugate paralyses clearly points to the region of the sixth nucleus of the same side as affected.

5. Paralysis of up or down motions, or both motions, indicate disease in the region of the corpora quadrigemina, but may be due to disease in the third nerves proper at the point of exit.

6. Reasoning from analogy, paralysis of convergence points to disease in the central gray below the aqueduct, but as yet autopsies are lacking.

7. Isolated paralysis of parts of a third nerve strongly suggests central disease, but is not proof of it.

8. A majority of the cases of eye paralysis occur in the syphilitic.

9. A paralysis which changes rapidly, quickly showing fatigue, is probably central in origin.

10. Transitory paralysis in the syphilitic is strongly suggestive of future tabes.

11. An eye paralysis, however simple it may seem, is always a just cause for suspicion of trouble to come, and demands a prompt and thorough examination of the patient.

12. There is no evidence that there is any form of connection between the sixth nucleus and the third, except in the cerebrum.

